# Causal Shannon–Fisher Characterization of Motor/Imagery Movements in EEG

**DOI:** 10.3390/e20090660

**Published:** 2018-09-02

**Authors:** Román Baravalle, Osvaldo A. Rosso, Fernando Montani

**Affiliations:** 1 IFLYSIB, CONICET & Universidad Nacional de La Plata, La Plata 1900, Argentina; 2 Departamento de Física, Facultad de Ciencias Exactas, UNLP Calle 49 y 115. C.C. 67, La Plata 1900, Argentina; 3 Departamento de Informática en Salud, Hospital Italiano de Buenos Aires & CONICET, Ciudad Autónoma de Buenos Aires C1199ABB, Argentina; 4 Instituto de Física, Universidade Federal de Alagoas (UFAL), Maceió 57072-900, Brazil; 5 Complex Systems Group, Facultad de Ingeniería y Ciencias Aplicadas, Universidad de los Andes, Santiago 12455, Chile

**Keywords:** EEG signals, brain oscillation patterns, bandt and pompe methodology, Fisher information and Shannon entropy, 02.50.-r, 05.45.Tp, 87.19.La

## Abstract

The electroencephalogram (EEG) is an electrophysiological monitoring method that allows us to glimpse the electrical activity of the brain. Neural oscillations patterns are perhaps the best salient feature of EEG as they are rhythmic activities of the brain that can be generated by interactions across neurons. Large-scale oscillations can be measured by EEG as the different oscillation patterns reflected within the different frequency bands, and can provide us with new insights into brain functions. In order to understand how information about the rhythmic activity of the brain during visuomotor/imagined cognitive tasks is encoded in the brain we precisely quantify the different features of the oscillatory patterns considering the Shannon–Fisher plane H×F. This allows us to distinguish the dynamics of rhythmic activities of the brain showing that the Beta band facilitate information transmission during visuomotor/imagined tasks.

## 1. Introduction

The problem of finding a thesaurus for brain activity is a central issue in cognitive sciences. This involves the coding and decoding of the brain activity, and, in some way, the search for the intrinsic “language” of the brain. This issue is important not only at the theoretical level, but also for its potentials applications. For instance, there exists communication systems named brain–computer interfaces (BCI) that allows a direct communication pathway between an enhanced or wired brain and an external device [1,2,3,4,5,6,7,8,9,10,11,12]. This kind of technology systems generates a new communication channel between the brain and a computer. For two reasons, the way of BCI communication cannot be completely arbitrary. First, the BCI use allows the user to dispatch specific directives to computers through brain signals alone [11]. Second, the brain signals are measured taking advantage of electroencephalography (EEG), and thereafter they are handled by the BCI [11].

A useful tool for detecting and understanding brain communication is EEG. EEG signals depict the electrical activity that takes place at the cortex, and provide an important instrument for understanding a variety of cognitive processes [13]. The EEG are the product of synchronized activity of the brain and variations in EEG oscillations patterns reflect the underlying changes in neuronal synchrony. Different brain activity patterns are related to attention. Measures of the relative contribution of EEG oscillations are particularly useful to investigate the emergent properties of the rhythmic activities of the brain [13]. The electrical activity of the brain can be considered chaotic and ruled by a nonlinear dynamics, therefore extracting information from this can be a difficult issue. Hence, we need to use measures that could account for the intrinsic dynamics of chaotic and highly nonlinear systems [13,14,15,16].

Let us emphasize that there is no theoretical basis for selecting which signal feature is the most useful for BCI communication [1,2,3,4,5,6,7,12,14]. In this sense, gaining further understanding of how information is encoded in the brain is very important. For instance, studies have shown that particular imagined tasks, for example hand movements, have measurable consequences on critical brain signals. This quality can be used for communicate intentions simply using imagery movements. Importantly, human subjects exhibit considerable oscillations in the Alpha1 ∈[8,10) Hz and Alpha2 ∈[10,13) Hz bands of the EEG recorded over sensorimotor areas when they are not doing any motor action, in sensory processing, or in imaginations of these actions or processing. During several years, these rhythms (called mu rhythms) remained undetected in many human subjects, but computer-based analyses made possible to find out their presence in most of the subjects. The mu rhythm is also associated also with the Beta rhythms (Beta1 ∈[13,18) Hz and Beta 2 ∈[13,31) Hz). While some of these beta rhythms are harmonics of mu rhythms, some are separable by topography and/or timing from mu rhythms, so they are independent EEG features. Other useful tool for detecting important features of brain signals, and that also can be used for BCI, is the electrocorticography (ECoG), which is less noisy and has more bandwidth than EEG. It has been found through these techniques that the mu/beta rhythms also decreases with motor or imagery motor tasks [1,2,3,4,5,6,7]. Furthermore, the activity in the gamma range (Gamma 1 ∈[31,41) Hz and Gamma 2 ∈[41,50) Hz) has been found to increase during this experimental conditions [1,2,3,4,5,6,7]. With some exceptions, task-related changes in these band are difficult to be detected in the EEG signals, mainly because these higher frequencies are noisier and more affected by muscular artifacts than the other bands.

Movement or preparation for movement, and also motor imagery, changes the mu and beta rhythms. These tasks produces a decrease of these rhythms. Because the human subjects can change these rhythms without engaging in actual movements, these rhythms could serve as the basis for a BCI [8,9,10,11]. They have an easy implementation, the lowest cost and ubiquity among health professionals, and therefore the EEG is a preferred tool to measure brain activity. And in this paradigm, the study of the brain rhythms are fundamental, mainly because these rhythms are, in some sense, the “language” we can detect with this technique. In this paper, thus, we use subtle measures coming from Information Theory to quantify the intrinsic dynamics of these rhythms. We focus on the Fisher Information measures, *F*, that are of central importance for an assessment of the accuracy of the relevant signal features (or parameters) being estimated. Importantly, the use of this Fisher permutation measure in combination with the permutation entropy, *H*, allows us to define the H×F causality information plane [17,18,19,20,21] a very useful tool to determine the more relevant rhythms, and negligible ones, for the particular experimental setup being analyzed in this paper.

## 2. Methodology

**Figure 1:**
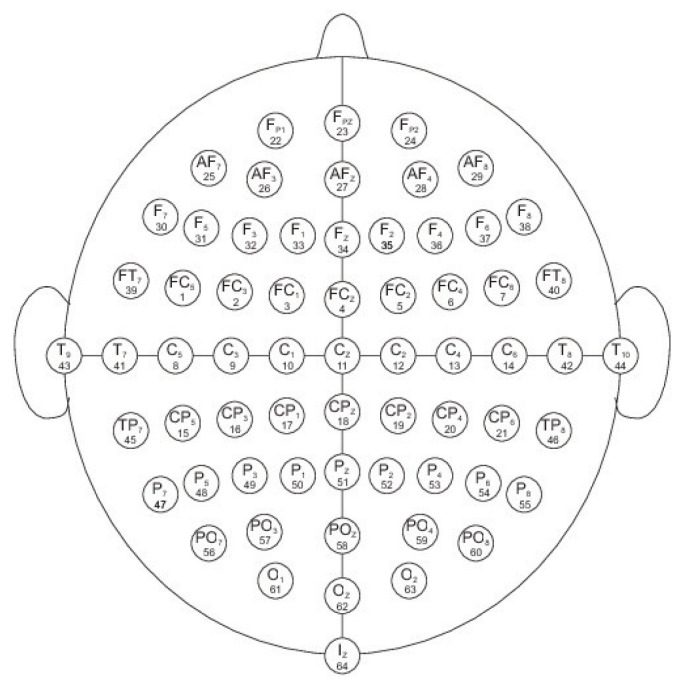
64 electrodes used to record the electrical activity of the brain recorded through the EEG signals (as in [11]).

The experimental setup of the BCI2000 system [11,22] is shown in Figure 1 that includes an arrangement of 64 electrodes used to record the electrical activity of the brain recorded through the EEG signals while the subjects perform different tasks, of visuomotor or imagery characteristics.

The electrical signals from the brain are picked up by small electrodes that are placed on the subject’s head. The electrodes only record the electrical activity of the brain and importantly they do not inject electrical current into the brain. The electrodes record the electrical activity from small areas of the brain.

The EEG bands allows us to gain further insights about the brain function, and look for the presence or absence of specific brain signals in specific areas of the brain. However, EEG raw signals by itself do not say anything of how the brain functions relate to the mind or which kind of information is carried by the different rhythmic oscillations. That is, we need to perform the EEG data analysis within an adequate theoretical framework that could allow us to gain further understanding of how brain functions are related to the mind.

### 2.1. Experimental Protocol

We considered the EEG Motor Movement/Imagery Dataset recorded using BCI2000 instrumentation system available through Physionet [23]. The dataset consists of more than 1500 EEG recordings, with different duration (one or two minutes per record), obtained from 109 healthy subjects. The 109-subjects data on Physionet is screening data in which the subjects had to perform one movement or imagery task at a time. The population of volunteers was drawn from the employer at the New York State Department of Health, i.e., all the subjects had completed high school training and most of them had completed four years of College education. The sex and age of the subjects have not been reported as relevant for performing these tasks. All subjects were naive. As learning proceeded with further repetitions of the experiment, imagery usually became less important and performance becomes more automatic [22,23,24,25,26,27]. Subjects were asked to perform different motor/imagery tasks while EEG signals were recorded from 64 electrodes along the surface of the scalp. Each subject performed 14 experimental runs:

- A one-minute baseline runs (with eyes open)
- A one-minute baseline runs (with eyes closed)
- Three two-minute runs of each of the four following tasks:
  1. A target appears on either the left or the right side of the screen. The subject opens and closes the corresponding fist until the target disappears. Then the subject relaxes.
  2. A target appears on either the left or the right side of the screen. The subject imagines opening and closing the corresponding fist until the target disappears. Then the subject relaxes.
  3. A target appears on either the top or the bottom of the screen. The subject opens and closes either both fists (if the target is on top) or both feet (if the target is on the bottom) until the target disappears. Then the subject relaxes.
  4. A target appears on either the top or the bottom of the screen. The subject imagines opening and closing either both fists (if the target is on top) or both feet (if the target is on the bottom) until the target disappears. Then the subject relaxes

The 64-channels EEG signals were recorded according to the international 10–20 system (as seen in Figure 1). The sampling frequency of the EEG is 160 Hz. The order of the recordings are: Baseline with open eyes, Baseline with closed eyes, and then Task 1, Task 2, Task 3 and Task 4 successively until the 14 runs where completed. The right mastoid bone corresponds to ground (GND) and the right earlobe to references (REF) [22,23,24,26,27]. The impedance was kept under 10 kOhm. The data are raw data without any re-referencing. There was no EOG recording. The data are raw without any post-processing. A variety of sensors for monitoring brain activity were used. Muscular artifacts (electromyographic (EMG) signals) were carefully checked at the beginning of each recording, and verified throughout the recording. During the recordings light was dimmed, and the experiment was performed in a closed room, so it was minimized external sounds. As the learning proceeds, imagery usually becomes less important [22,23,24,25,26,27]. The timing from the event markers was of 1 s and they are stated in the dataset (see Reference [23]), the tasks order was block-randomized, in blocks of 8. For further details of the EEG data acquisition we refer the reader to References [22,23,24,26,27]. For each subject and for each task we perform an average of the signals, resulting in event related potential (ERP) associated with each task.

The sampling frequency of the EEG are 160 Hz. But because of the high frequency artifacts that blurred the EEG, and to remove fluctuations at DC level and increase the signal resolution, the records where filtered first between 1–50 Hz using a filter based on the Kaiser window developed by Belitski et al., [28], with sharp transition bandwidth (0.1 Hz), small passband ripple (0.05 dB), and high stopband attenuation (60 dB).

After this filtering, a bandpass filtering were made. The bands in consideration are stated in Table 1.

Each signal has 20,000 points, thus this large number of points guarantees a good statistics for the Bandt and Pompe estimations considering an embedding dimension D = 6 (see Section 2.3).

### 2.2. Shannon Entropy and Fisher Information Measure

Sequences of measurements (or observations) constitute the basic elements for the study of natural phenomena. In particular, from these sequences, commonly called time series, one should judiciously extract information on the underlying dynamical systems under study. We can define an Information Theory quantifier as a measure that is able to characterize some property of the probability distribution function (PDF) associated with these time series of a given row signal (i.e., EEG). Entropy, regarded as a measure of uncertainty, is the most paradigmatic example of these quantifiers.

Given a time series $X\left(t\right)\equiv \left\{x_{t};t=1,\cdots ,M\right\}$, a set of *M* measures of the observable X and the associated PDF, given by $P\equiv \{p_{j};j=1,\cdots ,N\}$ with $\sum_{j=1}^{N}p_{j}=1$ and *N* the number of possible states of the system under study, the Shannon’s logarithmic information measure (Shannon entropy) [29] is defined by

(1) $$S\left[P\right]\;=\;-\sum_{j=1}^{N}p_{j}\ln\left(p_{j}\right)\;.$$

This functional is equal to zero when we are able to predict with full certainty which of the possible outcomes *j*, whose probabilities are given by $p_{j}$, will actually take place. Our knowledge of the underlying process, described by the probability distribution, is maximal in this instance. In contrast, this knowledge is commonly minimal for a uniform distribution $P_{e}=\left\{p_{j}=1/N,\forall j=1,\cdots ,N\right\}$.

The Shannon entropy *S* is a measure of “global character” that is not too sensitive to strong changes in the PDF taking place in small region. This is not the case with the Fisher information measure [30,31]

(2) $$F\left[f\right]\;=\;\int \frac{|\vec{\nabla }{f\left(x\right)|}^{2}}{f(x)}\;dx\;,$$

which constitutes a measure of the gradient content of the distribution *f* (continuous PDF), thus being quite sensitive even to tiny localized perturbations.

The Fisher information measure can be variously interpreted as a measure of the ability to estimate a parameter, as the amount of information that can be extracted from a set of measurements, and also as a measure of the state of disorder of a system or phenomenon [31,32], its most important property being the so-called Cramer-Rao bound. It is important to remark that the gradient operator significantly influences the contribution of minute local *f*-variations to the Fisher information value, so that the quantifier is called a “local” one. Note that Shannon entropy decreases with skewed distribution, while Fisher information increases in such a case. Local sensitivity is useful in scenarios whose description necessitates an appeal to a notion of “order” [33,34,35]. The concomitant problem of loss of information due to the discretization has been thoroughly studied (see, for instance, [36,37,38] and references therein) and, in particular, it entails the loss of Fisher’s shift-invariance, which is of no importance for our present purposes. For Fisher information measure computation (discrete PDF) we follow the proposal of Dehesa and coworkers [39] based on amplitude of probability $f\left(x\right)=\psi {\left(x\right)}^{2}$ then

(3) $$F\left[\psi \right]\;=\;4\int \;{\left\{\frac{d\psi }{dx}\right\}}^{2}\;dx\;.$$

Its discrete normalized version (0≤F≤1) is now

(4) $$F\left[P\right]\;=F_{0}\;\sum_{i=1}^{N-1}{\left(\sqrt{p_{i+1}}-\sqrt{p_{i}}\right)}^{2}\;.$$

Here the normalization constant $F_{0}$ reads

(5) $$F_{0}\;=\;\left\{\begin{array}{cc} 1 & \;\mathrm{if}\;p_{i^{*}}=1\;\mathrm{for}\;i^{*}=1\;\mathrm{or}\;i^{*}=N\;\mathrm{and}\;p_{i}=0\;\forall i\neq i^{*}\\ 1/2 & \;\mathrm{otherwise} \end{array}\right.\;.$$

If our system lies in a very ordered state, we can think it describe by a PDF given by $P_{0}=\left\{p_{k}≅1;\;p_{i}≅0\;\forall \;i\neq k;\;i=1,\cdots ,N\right\}$ (with *N*, the number of states of the system) in consequence we have a Shannon entropy $S[P_{0}]≅0$ and a normalized Fisher’s information measure $F\left[P_{0}\right]≅F_{max}=1$. On the other hand, when the system under study is represented by a very disordered state, one can think this particular state is described by a PDF given by the uniform distribution $P_{e}=\left\{p_{i}=1/N\;\forall \;i=1,\cdots ,N\right\}$ we obtain $S\left[P_{e}\right]≅S_{max}$ while $F[P_{e}]≅0$. One can state that the general behavior of the Fisher information measure is opposite to that of the Shannon entropy [40].

### 2.3. The Bandt–Pompe Approach to the PDF Determination

The study and characterization of time series X(t) by recourse to Information Theory tools assume that the underlying PDF is given a priori. In contrast, part of the concomitant analysis involves extracting the PDF from the data and there is no univocal procedure with which everyone agrees. Almost ten years ago Bandt and Pompe (BP) introduced a successful methodology for the evaluation of the PDF associated with scalar time series data using a symbolization technique [41]. For a didactic description of the approach, as well as, its main biomedical and econophysics applications, see Reference [42].

The pertinent symbolic data are (i) created by ranking the values of the series and (ii) defined by reordering the embedded data in ascending order, which is tantamount to a phase space reconstruction with embedding dimension (pattern length) *D* and time lag τ. In this way it is possible to quantify the diversity of the ordering symbols (patterns) derived from a scalar time series.

Note that the appropriate symbol sequence arises naturally from the time series and no model-based assumptions are needed. In fact, the necessary “partitions” are devised by comparing the order of neighboring relative values rather than by apportioning amplitudes according to different levels. This technique, as opposed to most of those in current practice, takes into account the temporal structure of the time series generated by the physical process under study. This feature allows us to uncover important details concerning the ordinal structure of the time series [35,43,44] and can also yield information about temporal correlation [17,18].

It is clear that this type of analysis of time series entails losing some details of the original series’ amplitude information. Nevertheless, by just referring to the series’ intrinsic structure, a meaningful difficulty reduction has indeed been achieved by Bandt and Pompe with regard to the description of complex systems. The symbolic representation of time series by recourse to a comparison of consecutive (τ=1) or nonconsecutive (τ>1) values allows for an accurate empirical reconstruction of the underlying phase-space, even in the presence of weak (observational and dynamic) noise [41]. Furthermore, the ordinal patterns associated with the PDF is invariant with respect to nonlinear monotonous transformations. Accordingly, nonlinear drifts or scaling artificially introduced by a measurement device will not modify the estimation of quantifiers, a nice property if one deals with experimental data (see, e.g., [45]). These advantages make the Bandt and Pompe methodology more convenient than conventional methods based on range partitioning (i.e., PDF based on histograms).

Additional advantages of the method reside in (i) its simplicity, we need few parameters: the pattern length/embedding dimension *D* and the embedding delay τ, and (ii) the extremely fast nature of the pertinent calculation process [46]. The BP methodology can be applied not only to time series representative of low dimensional dynamical systems, but also to any type of time series (regular, chaotic, noisy, or reality based). In fact, the existence of an attractor in the *D*-dimensional phase space is not assumed. The only condition for the applicability of the Bandt–Pompe methodology is a very weak stationary assumption (that is, for k≤D, the probability for $x_{t}<x_{t+k}$ should not depend on *t* [41]).

To use the Bandt and Pompe [41] methodology for evaluating the PDF, *P*, associated with the time series (dynamical system) under study, one starts by considering partitions of the pertinent *D*-dimensional space that will hopefully “reveal” relevant details of the ordinal structure of a given one-dimensional time series $X\left(t\right)=\left\{x_{t};t=1,\cdots ,M\right\}$ with embedding dimension D>1 (D∈N) and embedding time delay τ (τ∈N). We are interested in “ordinal patterns” of order (length) *D* generated by $\left(s\right)\;\mapsto \;\left(\;x_{s-(D-1)\tau },\;x_{s-(D-2)\tau },\;\cdots ,\;x_{s-\tau },\;x_{s}\;\right)$, which assigns to each time *s* the *D*-dimensional vector of values at times s,s−τ,⋯,s−(D−1)τ. Clearly, the greater the *D*-value, is the more information on the past is incorporated into our vectors. By “ordinal pattern” related to the time (s) we mean the permutation $\pi =(r_{0},r_{1},\cdots ,r_{D-1})$ of [0,1,⋯,D−1] defined by $x_{s-r_{D-1}\tau }\;\leq \;x_{s-r_{D-2}\tau }\;\leq \;\cdots \;\leq \;x_{s-r_{1}\tau }\;\leq \;x_{s-r_{0}\tau }$. In order to get a unique result we set $r_{i}<r_{i-1}$ if $x_{s-r_{i}}=x_{s-r_{i-1}}$. This is justified if the values of $x_{t}$ have a continuous distribution so that equal values are very unusual. Thus, for all the D! possible permutations π of order *D*, their associated relative frequencies can be naturally computed by the number of times this particular order sequence is found in the time series divided by the total number of sequences.

Consequently, it is possible to quantify the diversity of the ordering symbols (patterns of length *D*) derived from a scalar time series, by evaluating the so-called permutation entropy, the permutation statistical complexity and Fisher permutation information measure. Of course, the embedding dimension *D* plays an important role in the evaluation of the appropriate probability distribution because *D* determines the number of accessible states D! and also conditions the minimum acceptable length M≫D! of the time series that one needs in order to work with reliable statistics [43].

Regarding to the selection of the parameters, Bandt and Pompe suggested working with 4≤D≤6 and specifically considered an embedding delay τ=1 in their cornerstone paper [41]. Nevertheless, it is clear that other values of τ could provide additional information. It has been recently shown that this parameter is strongly related, if it is relevant, to the intrinsic time scales of the system under analysis [47,48,49].

The Bandt and Pompe proposal for associating probability distributions to time series (of an underlying symbolic nature), constitutes a significant advance in the study of non linear dynamical systems [41]. The method provides univocal prescription for ordinary, global entropic quantifiers of the Shannon-kind. However, as was shown by Rosso and coworkers [34,35], ambiguities arise in applying the Bandt and Pompe technique with reference to the permutation of ordinal patterns. This happens if one wishes to employ the BP-probability density to construct local entropic quantifiers, like Fisher information measure, that would characterize time series generated by nonlinear dynamical systems.

The local sensitivity of Fisher information measure for discrete-PDFs is reflected in the fact that the specific “*i*-ordering” of the discrete values $p_{i}$ must be seriously taken into account in evaluating the sum in Equation (4). The pertinent numerator can be regarded as a kind of “distance” between two contiguous probabilities. Thus, a different ordering of the pertinent summands would lead to a different Fisher information value. In fact, if we have a discrete PDF given by $P=\{p_{i},i=1,\cdots ,N\}$ we will have N! possibilities for the *i*-ordering.

The question is, which is the arrangement that one could regard as the “proper” ordering? The answer is straightforward in some cases, histogram-based PDF constituting a conspicuous example. For such a procedure one first divides the interval [a,b] (with *a* and *b* the minimum and maximum amplitude values in the time series) into a finite number on nonoverlapping sub-intervals (bins). Thus, the division procedure of the interval [a,b] provides the natural order-sequence for the evaluation of the PDF gradient involved in Fisher information measure. In our current paper, we chosen for the Bandt–Pompe PDF the lexicographic ordering given by the algorithm of Lehmer [50], amongst other possibilities, due to it provide the better distinction of different dynamics in the Fisher vs Shannon plane (see [34,35]).

## 3. Results

Numerous studies have shown that EEGs allow subjects to use motor images to modulate the rhythmic activity of different bands and thus control a BCI system (we refer the reader to Reference [11]). That is the BCI systems use some especial characteristics of the brain signals to summarize them into actions that will control a given output (i.e., a robotic arm). Rhythmic neural activity within Beta frequency band is modulated when healthy subjects performs different assignments that involves realized or imagined movements. However, there are a many different rhythms of the neuronal activity that are dependent on the variety of sensory motor stimuli [13]. Cracking this neural code means inferring the feedback circuits of the neurons that contribute to cortical rhythmic activities, and understanding language and the communication among the neurons, how they synchronizes and transmit information through the neuronal network [51,52,53,54,55]. Changes in these rhythms can provide us important feedback of how information is transmitted across neuronal populations. Here we use EEG signals in combination with an information theoretical approach to investigate changes in oscillatory power while healthy human participants performed different tasks, of motor or imagery characteristics that were recorded using the BCI2000 system [11,22,23,24,26,27]. We use a versatile method to quantify the Causal Shannon Fisher information of the different oscillation bands of the EEG signals, taking advantage of a Bandt–Pompe symbolization that captures temporal causality features of the signal [17,18,19,20,21]. More specifically, we consider measures accounting for the causal structure of the EEG signals: the Shannon permutation entropy (*H*) and the Fisher information (*F*).

**Figure 2:**
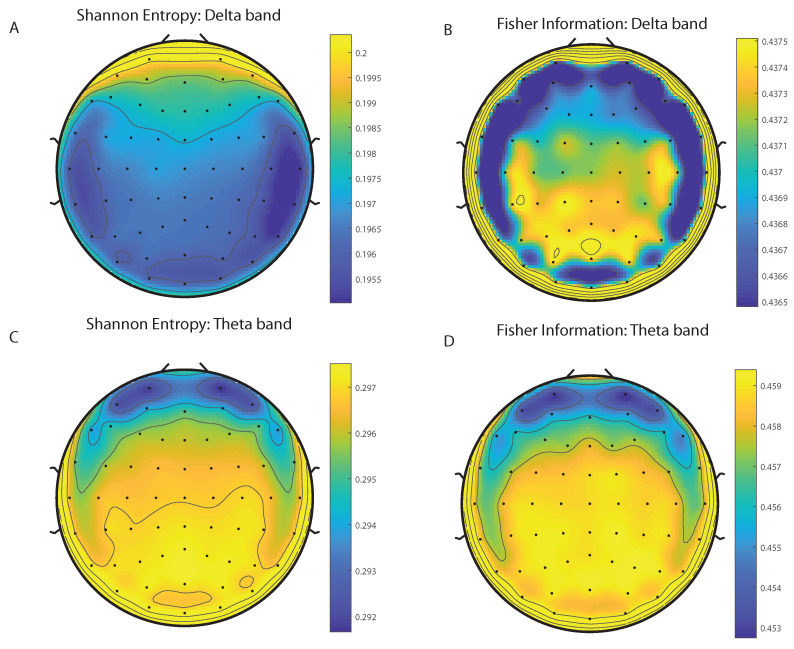
Averaged values of the normalized Shannon entropy and Fisher information, considering 109 subjects for the 64-channels of the EEG. (**A**) corresponds to the Shannon entropy for the Delta band and (**C**) to the Theta oscillation band, respectively. (**B**) corresponds to the Fisher information for Delta band and (**D**) to the Theta oscillation band, respectively. We consider D = 6 and τ = 1. We do not remove sample dependent bias from the Shannon Fisher estimations. The results are the same for the four different visuomotor/imagined tasks (the *t*-test does not rejects the null hypothesis in any of the cases under consideration).

**Figure 3:**
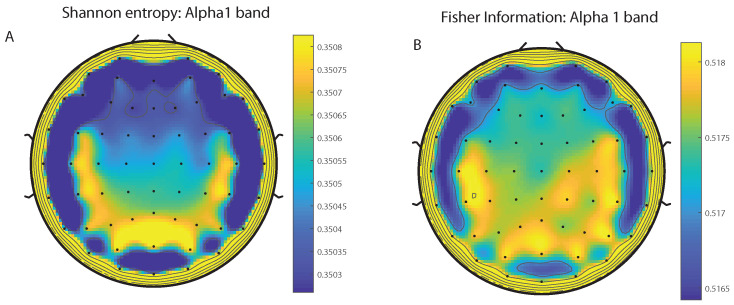

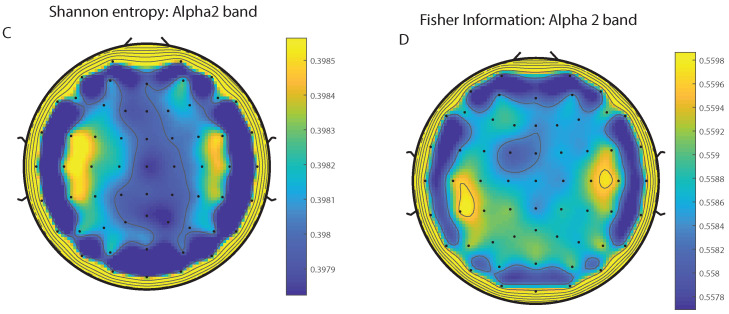
Averaged values of the normalized Shannon entropy and Fisher information, considering 109 subjects for the 64-channels of the EEG. (**A**) corresponds to the Shannon entropy for the Alpha 1 band and (**C**) to the Alpha 2 oscillation band, respectively. (**B**) corresponds to the Fisher information for Alpha1 band and (**D**) to the Alpha2 oscillation band, respectively. We consider D = 6 and τ = 1. We do not remove sample dependent bias from the Shannon Fisher estimations. The results are the same for the four different visuomotor/imagined tasks (the *t*-test does not rejects the null hypothesis in any of the cases under consideration).

**Figure 4:**
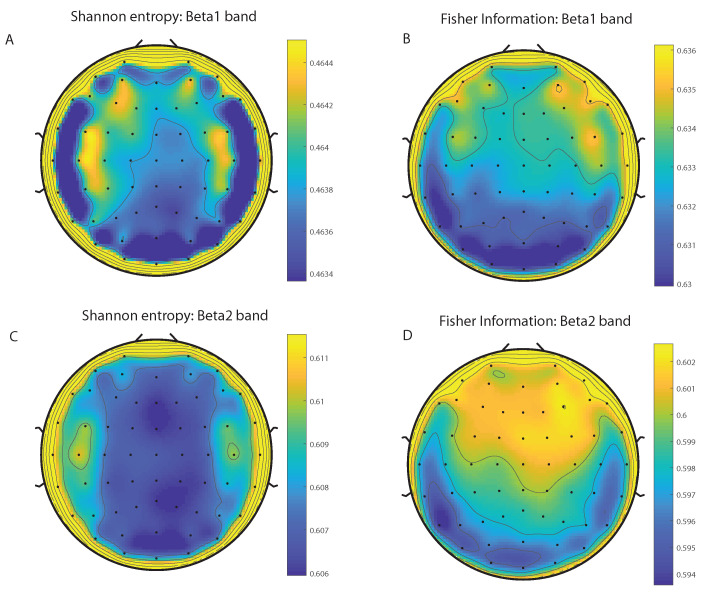
Averaged values of the normalized Shannon entropy and Fisher information, considering 109 subjects for the 64-channels of the EEG. (**A**) corresponds to the the Shannon entropy for the Beta 1 band and (**C**) to the Beta 2 oscillation band, respectively. (**B**) corresponds to the the Fisher information for the Beta 1 band and (**D**) to the Beta 2 oscillation band, respectively. We consider D = 6 and τ = 1. We do not remove sample dependent bias from the Shannon Fisher estimations. The results are the same for the four different visuomotor/imagined tasks (the *t*-test does not rejects the null hypothesis in any of the cases under consideration).

**Figure 5:**
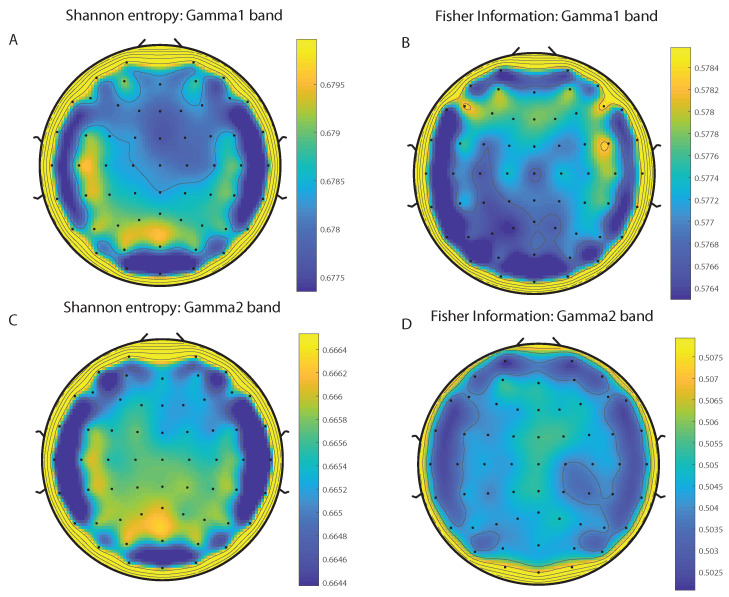
Averaged values of the normalized Shannon entropy and Fisher information, considering 109 subjects for the 64-channels of the EEG. (**A**) corresponds to the the Shannon entropy for the Gamma 1 band and (**C**) to the Gamma 2 oscillation band, respectively. (**B**) corresponds to the the Fisher information for the Gammma 1 band and (**D**) to the Gamma 2 oscillation band, respectively. We consider D = 6 and τ = 1. We do not remove sample dependent bias from the Shannon Fisher estimations. The results are the same for the four different visuomotor/imagined tasks (the *t*-test does not rejects the null hypothesis in any of the cases under consideration).

**Figure 6:**
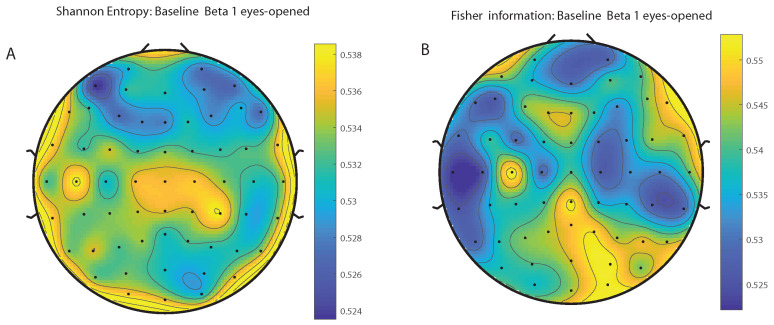

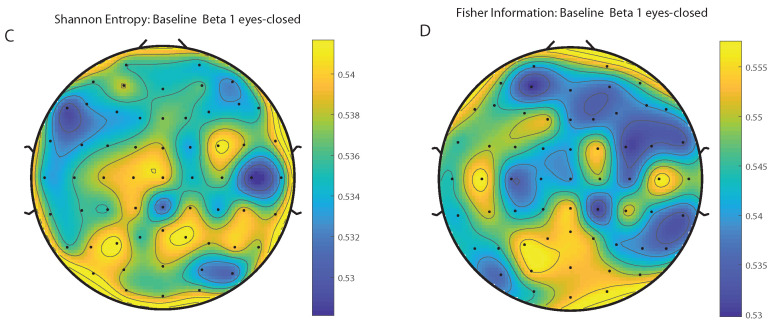
Averaged values of the normalized Shannon entropy and Fisher information, considering 109 subjects for the 64-channels of the EEG for the baselines signals. (**A**) corresponds to the the Shannon entropy for the Beta 1 band (eyes-opened) and (**C**) to the Beta 1 oscillation band (eyes-closed), respectively. (**B**) corresponds to the the Fisher information for the Beta 1 band (eyes-opened) and (**D**) to the Beta 1 oscillation band (eyes-closed), respectively. We consider D = 6 and τ = 1. We do not remove sample dependent bias from the Shannon Fisher estimations. The results are the same for the four different visuomotor/imagined tasks (the *t*-test does not rejects the null hypothesis in any of the cases under consideration).

**Figure 7:**
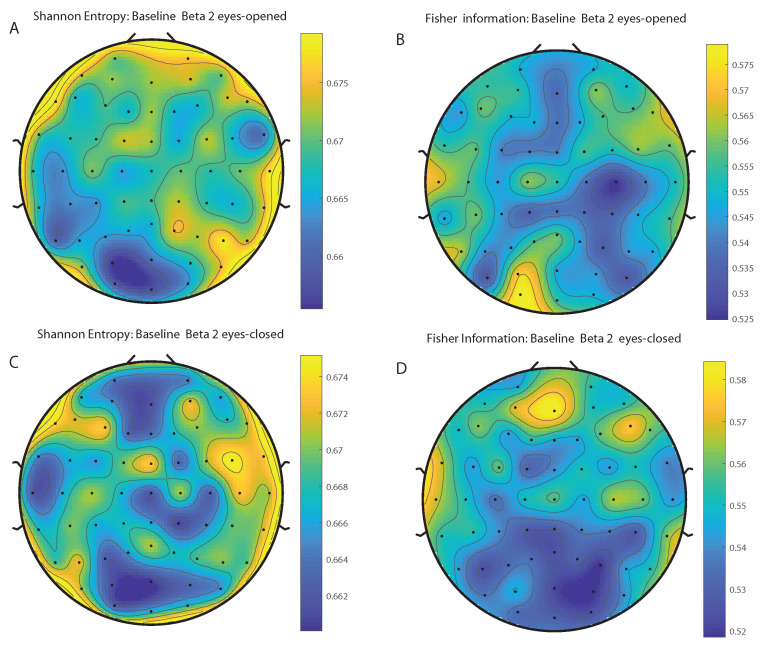
Averaged values of the normalized Shannon entropy and Fisher information, considering 109 subjects for the 64-channels of the EEG for the baselines signals. (**A**) corresponds to the the Shannon entropy for the Beta 2 band (eyes-opened) and (**C**) to the Beta 2 oscillation band (eyes-closed), respectively. (**B**) corresponds to the the Fisher information for the Beta 2 band (eyes-opened) and (**D**) to the Beta 2 oscillation band (eyes-closed), respectively. We consider D = 6 and τ = 1. We do not remove sample dependent bias from the Shannon Fisher estimations. The results are the same for the four different visuomotor/imagined tasks (the *t*-test does not rejects the null hypothesis in any of the cases under consideration).

We take advantage of the wICA methodology to remove all the muscular and technical artifacts of the EEG as it has been previously performed in References [56,57,58]. We use thereafter a filter based on the Kaiser window developed by Belitski et al., [28], to split the signal in the Delta, Theta, Alpha 1, Alpha 2, Beta 1, Beta 2, Gamma 1 and Gamma 2 oscillation bands (see Methodology for further details). In order to perform analyses within the BP formalism, we need to consider a large number of points of EEG responses (M≫D!). We have 20000 data points for each case that is enough signal to get a trustable estimation of Shannon Fisher information. In this case, we do not use the NSB methodology (see Reference [59]) to remove sample size dependent bias from H×F estimations due to the large amount of data available, and the intricacy of the methodology when performing the Fisher information estimations. We use the Bandt and Pompe [41] methodology for evaluating the PDF, *P*, associated with the time series, considering an embedding dimension D=6 and time lag τ=1. This embedding dimension (pattern length) is enough to efficiently capture the information causality of the ordinal structure of the time series [41]. Confidence error intervals cannot be provided within BP methodology. As we mentioned previously, the selection of the embedding dimension, *D*, is relevant for obtaining an appropriate probability distribution because *D* determines not only the number of accessible states (equal to D!) but also the length of the time series, *M*, needed to have a reliable statistics and therefore the requirement is that the condition M≫D! must be satisfied. Let us remark that as this condition is well satisfied the results presented in this section has been derived therefore without using a methodology to remove bias deviations from the entropy and Fisher information estimations. Figure 2A,C and Figure 3A,C show the averaged values of the normalized Shannon entropy considering 109 subjects for the 64-channels EEG considering the different oscillation bands Delta, Theta, Alpha 1 and Alpha 2, respectively. Figure 4A,C and Figure 5A,C depict the normalized Shannon entropy for the same conditions but taking the Beta 1, Beta 2, Gamma 1 and Gamma 2 bands, respectively. Shannon Entropy reach the maximum values within the Gamma 1 frequency band and the minimum values for the Delta oscillation band, respectively. In order to understand how is encoded the rhythmic activity of the brain during visuomotor tasks we estimate the Fisher information measure [17,18]. Figure 2B,D and Figure 3B,D show the averaged estimations of the Fisher information under the same conditions mentioned above for the Delta, Theta, Alpha 1 and Alpha 2 bands, respectively. Figure 4B,D and Figure 5B,D depict the Fisher information when considering the Beta 1, Beta 2, Gamma 1 and Gamma 2 bands, respectively. Notice that the Fisher information of the Beta 1 and Beta 2 oscillation bands is prevalent, while the Fisher information of the Delta band is significantly curtailed. Our results are the same for the four different visuomotor / imagined tasks. Let us remark that the Alpha 1 and Alpha 2 bands depict a significantly much lower amount of Fisher information than the Beta bands. The baselines for the Beta bands with open/closed eyes are presented in Figure 6 and Figure 7. Figure 6A,C and Figure 7A,C depict the Shannon entropy of the baselines when considering the Beta 1 and Beta 2 bands, respectively. Figure 6B,D and Figure 7B,D depict the Fisher information of the baselines when considering the Beta 1 and Beta 2 bands, respectively. Notice that the baselines present lower Fisher information values than the realized movements.

**Figure 8:**
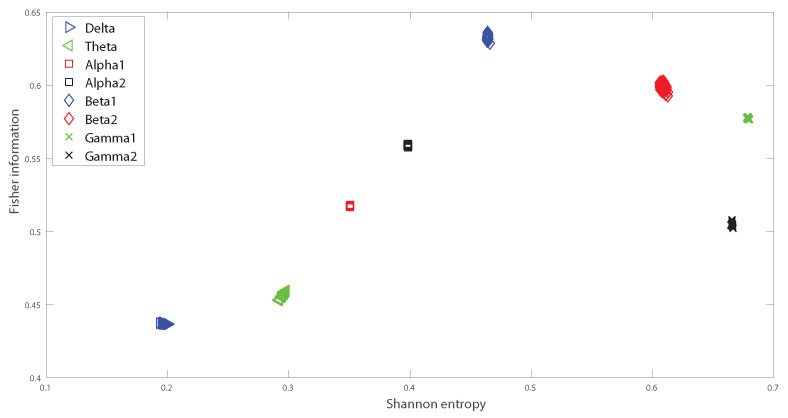
Shannon Fisher information. Fisher information versus normalized Shannon entropy (H×F-plane), averaging over all the subjects and each point is an electrode site, taking into account the different frequency bands. We consider D = 6 and τ = 1. We do not remove sample dependent bias from the Shannon Fisher estimations. The results are the same for the four different visuomotor/imagined tasks (the *t*-test does not rejects the null hypothesis in any of the cases under consideration).

**Figure 9:**
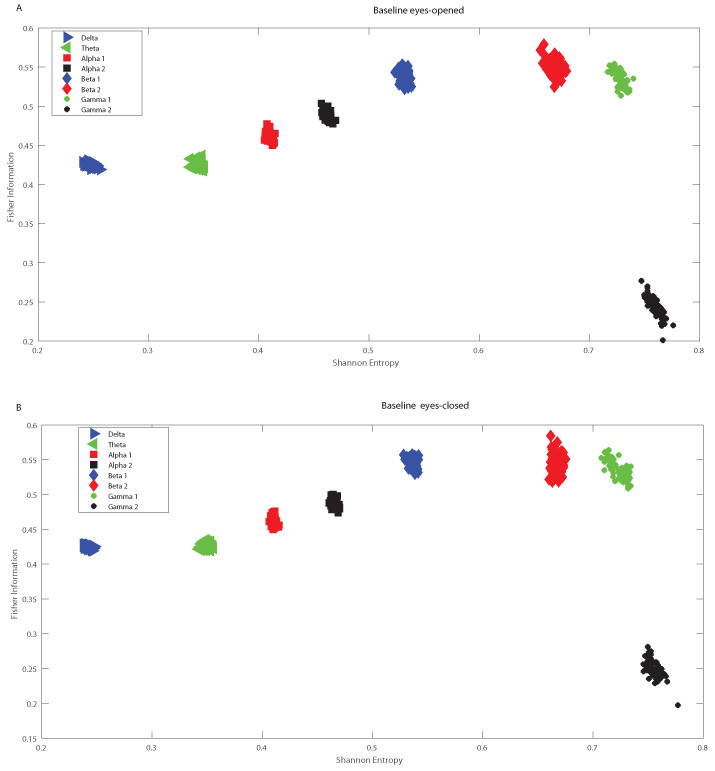
Shannon Fisher information for the baseline signals. Fisher information versus normalized Shannon entropy (H×F-plane) as in Figure 8 but considering the baseline signals. (**A**) corresponds to the eyes-opened. (**B**) corresponds to the eyes-closed.

In the following we analyze the localization of the different oscillation bands within the informational causal plane H×F, averaging over the 64 channels and taking into account all the subjects, considering the different frequency bands. The Fisher information provides important insights into how information is encoded in the different oscillation bands, and depends also on the structure of the brain. We observed some variations the H×F plane for the Beta bands, depending on the region of the brain in which the electrodes are located. However the Beta 1 band remains always as a maximum in H×F plane for the different electrodes, and therefore in Figure 8 we presented the averaged values. Figure 8 shows the mean over 109 subjects for the realized tasks, and considering the different electrodes. That is Figure 8 depicts the global behavior of the Shannon entropy-Fisher information oscillation bands across the brain. The maximum of Fisher Information is reached by the Beta 1 band, followed by lower values of the Beta 2, Alpha 1 and Alpha 2 bands. As the Beta-band is directly related to the disinhibition of neuronal populations involved in the computations of movement parameters, there are therefore just a few frequencies that are the preponderant ones inducing a maximal value for Fisher information. Thus Fisher information provides important additional information regarding the peculiarities of the underlying probability distribution function, that is not detected by the entropy. Notice that the combination of both, entropy and Fisher information, depicted in the plane H×F, allows us to distinguish the dynamics of different rhythmic oscillations bands. Importantly, Figure 9A,B depict the baselines with open and closed eyes, respectively (taking the mean over 109 subjects, and considering all the different electrodes). A simple comparison between the realized tasks with the baselines shows that Fisher information takes always higher values for realized task than for the baselines. Let us emphasize that the *t*-test performed showed that the values of the Fisher Information for the realized tasks are different from that of the baselines. That is, the *p*-values of the *t*-test resulted in all cases to be lower than 0.05 for each subject when comparing the Fisher information of the realized task for each electrode with the corresponding baseline. Thus Beta band facilitates information transmission across when considering the visuomotor tasks. Let us recall that if the system is in a very ordered state and thus is represented by a very narrow PDF, we have a Shannon entropy close to zero and a Causal Fisher information measure closer to maximum value. On the other hand, when the system is in a very disordered state one gets an almost flat PDF and a Shannon entropy closer to the maximum, while the Causal Fisher information is equal to zero. One can state that the general behavior of the Fisher information measure is opposite to that of the Shannon entropy [40]. Thus the use of the normalized Shannon entropy in combination with the Causal Fisher information, we can identify the more informative oscillation bands that could be of help to decode certain movements/intentions to translate them into movements of artificial actuators.

## 4. Conclusions and Discussions

Developing new theoretical tools that could provide insights of how mind/brain mechanisms are related can crucially change our understanding of cognitive processes [56]. We have recently provided a methodology to estimate the statistical complexity of the different rhythmic activities of the brain removing bias deviations from the estimations through the NSB methodology [59]. However, when considering the case of the Causal Shannon Fisher information removing size sampling dependent bias is not an straightforward task as involves the gradient of the score function. We will consider this issue of removing sample size dependent bias from Shannon Fisher information through the NSB methodology in a future publication. Moreover, as the large number of electrode considered in this study might constrain a wider application of proposed BCI, in future research we will reduce the number of electrode presented here following the methodologies of References [60,61]. It would be also of interest to investigate in the close future event related synchronization and desynchronization to study the phase-locking underlying mechanism as in References [62,63]. In our current paper we show an application of the Shannon Fisher information to quantify how information is transmitted through the different oscillation bands. Importantly in this paper, we show that recent advances in Shannon Fisher information can provide crucial new insights into neuronal dynamics and how information is transmitted in the brain. We investigate then the Fisher information versus Shannon entropy plane, H×F considering that the EEG oscillations patterns reflect the underlying changes in neuronal synchrony as subjects performs a visuomotor or imagined cognitive tasks. We provide a causal mapping, H×F, of the dynamical rhythmic activities of the brain that might provide a measure of attentional investment. Importantly, the causal mapping H×F allows us to characterize the internal dynamics patterns of the brain, providing important insights how much information is encoded within the different brain oscillation bands.

We show that the maximum of the Fisher information is reached within the Beta oscillation band. However, notice that while Fisher information about the Beta oscillation band remains maximal, the Alpha bands reaches a much lower information values. This is consistent with recent findings showing that during visuomotor tasks there is an increase in the oscillatory power of the Alpha band with a correspondent decrease in oscillatory power occurred in the Beta band [64]. This attenuation in the oscillatory power leads to an attenuation in the strength of the signal or at least within the prevailing frequencies leading to maximal amount of Fisher information within the Beta oscillation band. Interestingly, the Beta and Alpha bands form part of the so called sensory motor/mu rhythm, which experiment a change when the subject do or imagine a movement, an thus in a way it expresses intention to move [64].

Alpha and Beta band oscillations are implicated in sensory anticipation and motor preparation. On one hand, the Beta-band is directly related to the disinhibition of neuronal populations involved in the computations of movement parameters and translated in a consequent reduction of the oscillatory power within this band, leading to a higher Fisher information. On the other hand, the Alpha band is an important piece for cognitive processes, it has been proved that it is involved in long range synchronizations between brain areas, and related to cognitive processes of visuospatial attention [65]. Importantly, the oscillatory power in the Alpha- and Beta-bands are highly “desynchronizated” [66,67]. There is indeed intriguing evidence that these rhythms serve distinct functions and support mental simulation of actions with different functional mechanisms [64]. Alpha- and Beta-band rhythms are functionally dissociated. An increase in the power of the Alpha band, it comes combined with a decrease of the Beta-band power.

Alpha and Beta band power modulations are usually manifested throughout an increased excitability of the Alpha band with a consequent suppression in Beta band [68]. Moreover, Alpha and Beta band desynchronization has been related to kinematic regularities [68]. The power attenuation of different rhythms is commonly used in brain machine interface research, i.e., to decode fine movement properties using noninvasive techniques. In this paper we use the plane Shannon entropy versus Fisher information, H×F, that allows us to distinguish the dynamics of different rhythmic oscillations bands during visoumotor tasks. That is the Fisher Information can be used as a biomarker of which bands varies more during a given task, making it a valuable tool for a BCI system. After the analysis in the H×F plane, one could concentrate more in the bands that are more informative in the particular experiment. In this sense, the causal Fisher Information is a valuable tool, because it provides insight in the system, and also is very easy to estimate. The causality Shannon entropy-Fisher information plane H×F could serve as the basis for a successful BCI, a technology that can help people who are severely disabled connected to a noninvasive EEG device. Humans can use motor imagery to modulate activity in the Beta or Alpha bands, and to thereby control a brain computer interface (BCI) system. The possibility of inferring motion kinematics based on noninvasive neural signals may be of great importance for developing and improving existing brain machine interface technologies to be used for motor rehabilitation [12,69]. This is to say that by revealing different characteristics of the rhythmic oscillations patterns within the Causal Shannon–Fisher information plane, H×F, one would tell which specific signal sequence would be of mean for certain functions that could be decoded to the artificial actuators like a robotic arm.

## Figures and Tables

**Table 1:** Frequency bands analysed.

Band	Frequency Interval (Hz)
Delta	[1,4)
Theta	[4,8)
Alpha 1	[8,10)
Alpha 2	[10,13)
Beta 1	[13,18)
Beta 2	[18,31)
Gamma 1	[31,41)
Gamma 2	[41,50)
